# Financial Protection vs Autonomy, Perspectives from Fraud Victims on A Financial Account Temporary Hold Policy

**DOI:** 10.1093/geroni/igaf122.3141

**Published:** 2025-12-31

**Authors:** Louise (Siyu) Gao, Marguerite DeLiema, Sohum Bindra

**Affiliations:** University of Minnesota, Minneapolis, Minnesota, United States; University of Minnesota School of Social Work, Minneapolis, Minnesota, United States; University of Minnesota Twin Cities, Minneapolis, Minnesota, United States

## Abstract

A temporary financial account hold policy, also known as an authorization hold, is a temporary restriction on transferring funds out of a bank or brokerage account. This policy may be utilized as a consumer protection tool to prevent older adult accountholders from elder financial exploitation. Our qualitative study interviewed 12 older fraud victims or their authorized proxies (mean age: 75; mean loss amount: $52,690 ranging from $3500 to $240,000) who experienced a temporary account hold to understand the benefits, costs, and the psychological impact. More than half of the participants showed appreciation and understood the intent of their financial institution(s) to keep them safe from fraud or abuse. However, some perceived the protective temporary hold as infringing on their right to financial self-determination and were upset. A minority of participants faced negative economic impacts, such as credit card companies closing victims’ accounts due to risk concerns, difficulty opening new accounts elsewhere, credit score drops, and inability to access their funds for daily activities. Psychosocial outcomes included feeling anxious, depressed, ashamed, and lonely. Participants recommended more transparency, communication, emotional support, and education. Using thematic analysis, this study will offer victims’ perspectives on the impact of the temporary account hold policy and facilitate a critical discussion on the importance of self-determination in the consumer protection field. Victims’ stories of financial exploitation and their attitudes toward perpetrators will also be discussed.

